# Adult Attachment Orientations and Social Networking Site Addiction: The Mediating Effects of Online Social Support and the Fear of Missing Out

**DOI:** 10.3389/fpsyg.2019.02629

**Published:** 2019-11-26

**Authors:** Chang Liu, Jian-Ling Ma

**Affiliations:** ^1^Yangtze Normal University, Chongqing, China; ^2^Chongqing University of Posts and Telecommunications, Chongqing, China

**Keywords:** adult attachment, anxious attachment, fear of missing out, online social support, social networking site addiction

## Abstract

Evidence supports predictive roles of adult attachment orientations for the maintenance of social networking site (SNS) addiction, but the underlying mechanisms are mostly unknown. Based on attachment theory, this study explored whether online social support and the fear of missing out mediated the relationship between insecure attachment and social networking site addiction among 463 college students in China. A questionnaire was used to collect data using the Experience in Close Relationship Scale—Short Form, online social support scale, fear of missing out scale, and Chinese Social Media Addiction Scale. The results showed that online social support and fear of missing out mediated the relationship between anxious attachment and social networking site addiction in parallel paths and serially, and online social support negatively mediated the relationship between avoidant attachment and social networking site addiction. Theoretically, the present study contributes to the field by showing how insecure attachment is linked to SNS addiction. Practically, these findings could aid in future studies on SNS addiction prevention and interventions. Limitations of the present study were discussed.

## Introduction

A social networking site (SNS) has been defined as an online service platform or site that focuses on facilitating the building of social networks and social relations among people who share interests, activities, backgrounds, or real-life connections (Wikipedia, 2012). It enables us to communicate, send posts, comment, and other social interactions among close, intimate, acquaintances, and strangers. Although SNSs have considerable benefits, excessive use of SNS has been found to have detrimental effects on human behavior (Marino et al., 2018a). Therefore, many scholars have explored factors associated with SNS addiction, among which attachment has received much attention (Blackwell et al., 2017; Monacis et al., 2017; Flynn et al., 2018; Worsley et al., 2018a; Chen, 2019; D’Arienzo et al., 2019; Marino et al., 2019). However, the underlying mechanisms between attachment and SNS addiction remain unclear. The present study aimed to explore the possible mediating roles of online social support and “the fear of missing out” (FOMO) in the association between attachment and SNS addiction. Identifying the mechanism underlying this association may further aid researchers in understanding why some people develop problematic SNS use and help in the development of effective prevention and intervention measures for problematic SNS use.

SNS addiction has been defined as an excessive concern about social media, being driven by a motivation to use social media, and devoting so much time and effort to social media that it limits other social activities, studies, interpersonal relationships, mental health, and well-being (Kuss and Griffiths, 2011, 2017; Griffiths, 2013). Although it has not been acknowledged as a formal disorder, researchers have already made efforts to clarify this relatively new behavior. In the present study, we applied attachment theory to SNS addiction to explain why differences in adult attachment could contribute to this phenomenon.

Adult attachment theory posits that individual relationship in adulthood originates from social interactions in infancy experienced with primary caregivers. According to Bowlby, the earliest emotional bonds individuals formed their caregivers have a during impact that continues throughout life (Bowlby, 1969/1982, 1973, 1980). These experiences are the basis of the internal working model, which is the mental representation of the self and others. This model is relatively stable and carries into adulthood, thereby forming the basis of interpersonal relationships (Hazan and Shaver, 1987; Feeney and Noller, 1990; Bartholomew and Horowitz, 1991). When a caregiver is perceived as responsive, accessible, and trustworthy, an infant develops a secure attachment. However, when the primary caregiver is inconsistent, unavailable, and/or unresponsive, a negative internal working model and an insecure attachment can develop (Bretherton, 1987). While attachment orientations displayed in adulthood are not necessarily the same as those seen in infancy, research indicates that early attachments can have serious impacts on later relationships. There are two dimensions of insecure attachments in adults: attachment anxiety and avoidance (Brennan et al., 1998). Anxious attachment (i.e., having high attachment anxiety) is characterized by a hyperactivate attachment system, which leads to a constant need to seek support and comfort. Conversely, avoidant attachment is characterized by an underactive attachment system, which leads to continual inhibition of psychological and social relationship needs, excessive self-reliance, and a marked distance from others.

Prior studies found that anxious attachment dimension positively predicted SNS addiction, while avoidant attachment dimension negatively predicted SNS addiction (Blackwell et al., 2017; Worsley et al., 2018a). Such an association is reasonable. In essence, SNSs are platforms for establishing and maintaining social relationships in cyberspace. Individuals with high level of anxious attachment fear that they will be abandoned; thus, they use SNS excessively to seek attention and comfort. Avoidant attachment doubted the effectiveness of available support and believes that support from their partner will not help them feel better. Overly, they deny their desire for closeness and intimacy. To further understand, in light of differences in attachment, why some people use SNS problematically, it is necessary to consider some crucial mediating variables such as online social support and FOMO.

Based on the above analysis, we formed our first hypothesis: attachment anxiety positively predicts, while attachment avoidance negatively predicts, SNS addiction (H1).

Social support is usually defined as help offered by someone with whom the recipient has an interpersonal relationship, including family members, friends, colleagues, or significant others (Zimet et al., 1988). Online social support refers to the support individuals who obtain through online settings. Online social support has become more important in the modern age of computers, especially since the emergency of SNS. Compared to offline social support, the online social support provided by SNS has several unique features. Examples of how emotional support is provided by SNS include “likes” and “favorites,” described as paralinguistic digital affordances (PDAs), which are “cues in social media that facilitate communication and interaction without specific language associated with their messages” (Kim, 2014; Hayes et al., 2016). In addition, it has been found that instrumental support, informational support, network management (Lin et al., 2016), perceived support (Lu and Hampton, 2016), and enacted support are all provided through SNS. Moreover, online social support provided through SNS is positively linked with the continued use of SNS and is also a significant predictor of SNS addiction (Lin et al., 2018; Liu and Ma, 2018b; Brailovskaia et al., 2019).

There is ample evidence that attachment affects individual differences in perceptions of social support in offline settings (Florian et al., 1995; Priel and Shamai, 1995; Anders and Tucker, 2000; Rholes et al., 2001). Adults with secure attachment are often confident that social support is available to them, and they are satisfied with the social support they receive. By contrast, adults with insecure attachment report receiving less social support and being less satisfied with the social support they do receive. However, the association between adult attachment orientations and perceptions of online social support may be different. Prior studies found that individuals with an anxious attachment attempt to seek social support and comfort online (Hart et al., 2015). They often worry that their friends will abandon them, and they try to be as emotionally close as possible to others. These individuals are, relatively, more sensitive to feedback and express more feedback-seeking behaviors on SNS than others (Hart et al., 2015). Individuals with an avoidant attachment dimension, however, generally deactivate their attachment systems to inhibit their psychological needs by expressing self-reliance, maintaining an emotional distance from others, and suppressing their needs for social support, such as likes, comments, and posts from others through SNS.

Based on our review of the relevant literature, we hypothesized that attachment anxiety would positively predict a need for online social support and a tendency toward SNS addiction, while attachment avoidance would negatively predict the need for online social support. In other words, the need for online social support mediated relationship between the insecure attachment and SNS addiction (H2).

FOMO is a recent phenomenon attracting scholarly attention as the prevalence of SNS increases. It refers to the feeling that others may be having rewarding experiences from which one is absent, characterized by the desire to stay connected with what others are doing (Przybylski et al., 2013). Thus, FOMO could act as both a predictor of social media engagement and addiction (Alt, 2015; Elhai et al., 2016) and a mediator between individual differences and SNS addiction (Beyens et al., 2016; Dempsey et al., 2019).

Indirect and direct evidence supports the idea that attachment differences may be correlated with FOMO. Studies have found that attachment is linked to psychological needs, which are correlated with FOMO. Specifically, both attachment anxiety and avoidance have been related to satisfying basic psychological needs (LaGuardia et al., 2000; Wei et al., 2005; Felton and Jowett, 2013; Miner et al., 2014). Other studies found that both anxious and avoidant attachment dimensions positively predicted basic psychological need satisfaction (Wei et al., 2005). That is the higher level of insecure attachment, the higher level of basic psychological needs frustration. Recently, a study conducted based on Facebook found that anxious attachment negatively correlated with the basic psychological need of “relatedness” (Lin, 2016; Chen, 2019). Furthermore, research showed that frustration with psychological needs led to FOMO (Przybylski et al., 2013; Xie et al., 2018). Self-determination theory proposes three intrinsic individual needs: competence, relatedness, and autonomy (Ryan and Deci, 2000). If these needs are not properly satisfied, it can develop maladaptive behaviors. In social media settings, individuals whose basic psychological needs are not satisfied are more likely to experience FOMO (Przybylski et al., 2013). Furthermore, a study found that both attachment anxiety and avoidance positively influenced FOMO (Blackwell et al., 2017), but this study did not address the reasons for those relationships. Overall, these studies indicate that the higher one’s level of attachment anxiety, the more one experiences FOMO.

Based on this analysis, we hypothesized that attachment anxiety and avoidance would positively predict FOMO, while the attachment anxiety would positively predict SNS addiction. In other words, FOMO mediates the relationship between insecure attachments and SNS addiction (H3).

**Figure 1:**
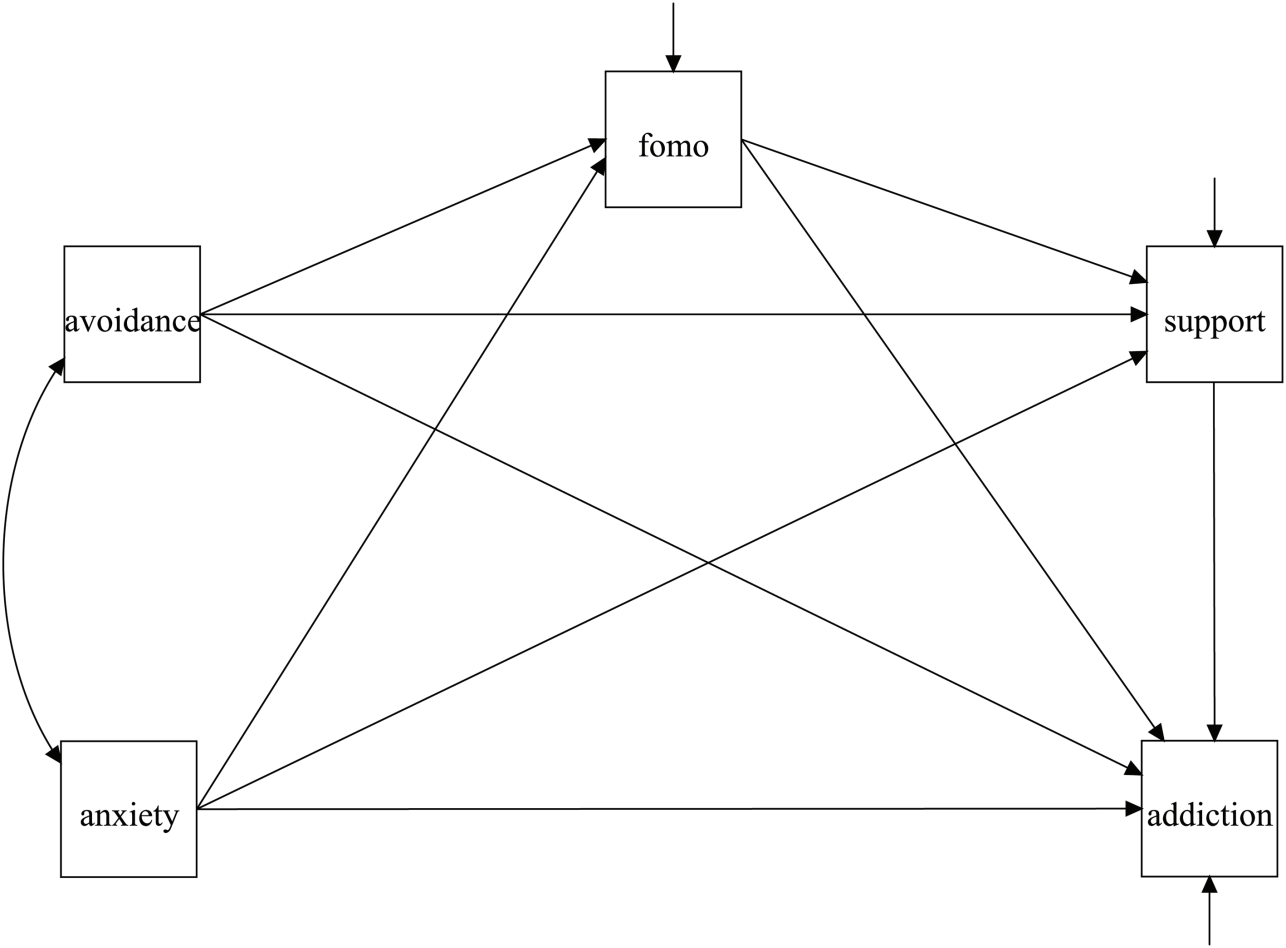
The hypothesized model of present study.

Based on our literature review, we theorized that online social support and FOMO play mediating roles in the link between insecure attachment and SNS addiction. To yield further insights into the exact association between insecure attachment and SNS addiction among Chinese college students, this study adopted an integrated multiple mediation model capable of simultaneously examining multiple influencing mechanisms of insecure attachment on SNS addiction. It has been suggested that a multiple mediation model could explore the complex mechanisms of consequent variables, making the model a crucial tool for improving the accuracy and applicability of theories (Hayes, 2017). Under the sequential mediation model, online social support and FOMO may exert a sequential mediating effect on the link between attachment avoidance, anxiety, and SNS addiction, with avoidance and anxiety attachment first giving rise to higher FOMO, which in turn promotes a higher need for online social support. Thus, insecure attachment can be viewed as a predictor for SNS addiction. Existing research has provided indirect evidence for this sequential mediation model. Liu and Ma (2018b) found that FOMO positively correlated with the need for online social support. Thus, we hypothesized that FOMO could positively predict the need for online social support. That is, FOMO and online social support serial mediated the relationship between insecure attachment and SNS addiction (H4). The study’s conceptual model is shown in Figure 1.

## Materials and Methods

### Participants

A total of 463 college students in China (344 females) aged 17–24 years (*M* = 19.94, SD = 1.11) volunteered to participate in our study and completed a questionnaire. The questionnaire was designed to collect information on the dependent variable (SNS addiction), the key independent variables of experiences in close relationships (attachment dimensions), online social support, FOMO, and demographic characteristics. All materials and procedures used in the study were approved by the Ethics in Human Research Committee of Chongqing University of Posts and Telecommunications. Informed written consent was obtained from all participants who were told that their participation was voluntary and they could terminate their participation at any time. The participants received no form of reward.

### Measures

#### Adult Attachment Scale

Adult attachment orientation was measured using the Experience in Close Relationship Scale—Short Form (ECR-SV; Wei et al., 2007). The ECR-SV is a 12-item index on adult attachment orientations with responses rated on a seven-point Likert-type scale where 1 = *strongly disagree* through 7 = *strongly agree*. The 12 items are on two six-item subscales, one on anxious attachment and the other on avoidant attachment. The ECR-SV has demonstrated strong psychometric reliability and validity in Asian population samples (Lee and Shin, 2019, pp. 119–127). In the present study, Cronbach’s *α* = 0.84 for the anxious attachment items and *α* = 0.75 for the avoidant attachment items.

#### Online Social Support

Online social support was measured using the Chinese online social support scale (Liu and Ma, 2018b), which was adapted from a Facebook-based measure of social support (McCloskey et al., 2015). The Chinese version comprises three dimensions with three items for each: perceived support, emotional support, and informational/instrumental support. Cronbach’s *α* for the whole scale was 0.80 in the present study.

#### The Fear of Missing Out Scale

FOMO was measured using 10 items with responses rated on a five-point Likert-type scale where 1 = *not at all true of me* through 5 = *extremely true of me* (Przybylski et al., 2013). The scores on the items were summed to create a composite measure of the extent to which the respondents feared that they were missing out on experiences and others were perceived to have. Cronbach’s *α* in the present study was 0.83.

#### Chinese Social Media Addiction Scale

The Chinese Social Media Addiction Scale (Liu and Ma, 2018a) was used to measure the extent of SNS addiction. It comprises 28 items divided into six dimensions: preference for online social interaction (6 items), mood alteration (5 items), negative consequences and continued use (5 items), compulsive use and withdrawal (6 items), salience (3 items), and relapse (3 items). Responses were rated on a Likert scale where 1 = *strongly disagree* through 5 = *strongly agree* and higher scores indicated more SNS addiction. In the present study, Cronbach’s *α* for the whole scale was 0.94.

### Methods of Statistical Analysis

All data collected in this study were recorded on a computer and processed using SPSS 18.0 and M*plu*s 7.4. Data processing was carried out in four steps, in accordance with recent literature on multiple mediation analyses. First, a factor analysis was performed to conduct a common variance analysis for testing common method biases. Second, scores from the four questionnaires were analyzed using descriptive statistics and correlation analysis. Third, M*plu*s 7.4 was used to evaluate the mediation effects of FOMO and online social support. The mediation model was estimated using the bootstrapping method to produce 95% bias-corrected confidence intervals of the effects from 5,000 samples of the data. Confidence intervals that did not include zero were interpreted as statistically significant effects at *p* ≤ 0.05. Before mediation analysis, a *t* test was conducted to explore whether there were gender differences concerning four variables in response to the gender imbalance in the present study. Before four-step formal analysis was performed, a K-means cluster analysis was conducted to explore how many participants displayed problematic SNS use.

## Results

### Cluster Analysis

K-means cluster analysis was used to identify participants’ levels of SNS addiction. Cluster analysis is a statistical method that allows participants to be divided into groups based on their similarities. To examine SNS addiction, a series of K-means cluster analyses were performed for the SNS addiction scores. The analysis resulted in participants being assigned to three groups: ordinary user, risky user, and problematic user (addictive user). The number of participants in each group was 104, 240, and 119, respectively; thus, the prevalence of SNS addiction in present study is 25.7%.

### Common Variance Bias

Common variance analysis was applied to the four questionnaires through factor analysis. The *χ*^2^ statistic of Bartlett’s test of Sphericity was significant. After principal component analysis, 12 eigenvalues greater than 1 were extracted. The first factor explained 26.9% of the total variance, which was less than the 40% required by the critical standard (Podsakoff et al., 2003), demonstrating that the questionnaires used in the current study had no significant issues related to common method biases.

### Descriptive and Correlation Statistics

Participants’ descriptive statistics are presented in Table 1. Anxious attachment dimension was positively related to the need for online social support, FOMO, SNS addiction, and avoidant attachment (all *p* < 0.01). Moreover, anxious and avoidant attachment positively correlated with each other (*p* < 0.01), but the relationships between avoidant attachment and online social support, FOMO, and SNS addiction were not statistically significant.

**Table 1:** Descriptive statistics and results of correlational analysis of variables (*N* = 463).

	1	2	3	4	5
1. Attachment avoidance	1				
2. Attachment anxiety	0.17^**^	1			
3. Online social support	−0.04	0.31^**^	1		
4. FOMO	0.07	0.36^**^	0.37^**^	1	
5. SNS addiction	0.07	0.39^**^	0.51^**^	0.56^**^	1

** *Correlation is significant at the 0.01 level (two-tailed)*.

### Mediation Analysis

To explore gender differences relating to the variables, *t* tests showed gender differences for FOMO (*M*_male_ = 24.79, *M*_female_ = 20.79, *t* = 5.01, *p* < 0.001), need for online social support (*M*_male_ = 36.67, *M*_female_ = 34.68, *t* = 2.23, *p* < 0.05), and SNS addiction (*M*_male_ = 83.03, *M*_female_ = 76, *t* = 3.45, *p* < 0.01), but not for attachment avoidance (*M*_male_ = 21.85, *M*_female_ = 21.88, *t* = −0.06, *p* = 0.95) and anxious attachment (*M*_male_ = 22.58, *M*_female_ = 21.60, *t* = 1.86, *p* = 0.06). Thus, when we performed a mediation analysis, gender was added as a control variable. In this model, gender significantly predicted FOMO (*b* = 3.48, *p* < 0.001) but not the need for online social support (*b* = 0.35, *p* = 0.71) or SNS addiction (*b* = 1.17, *p* = 0.46).

**Figure 2:**
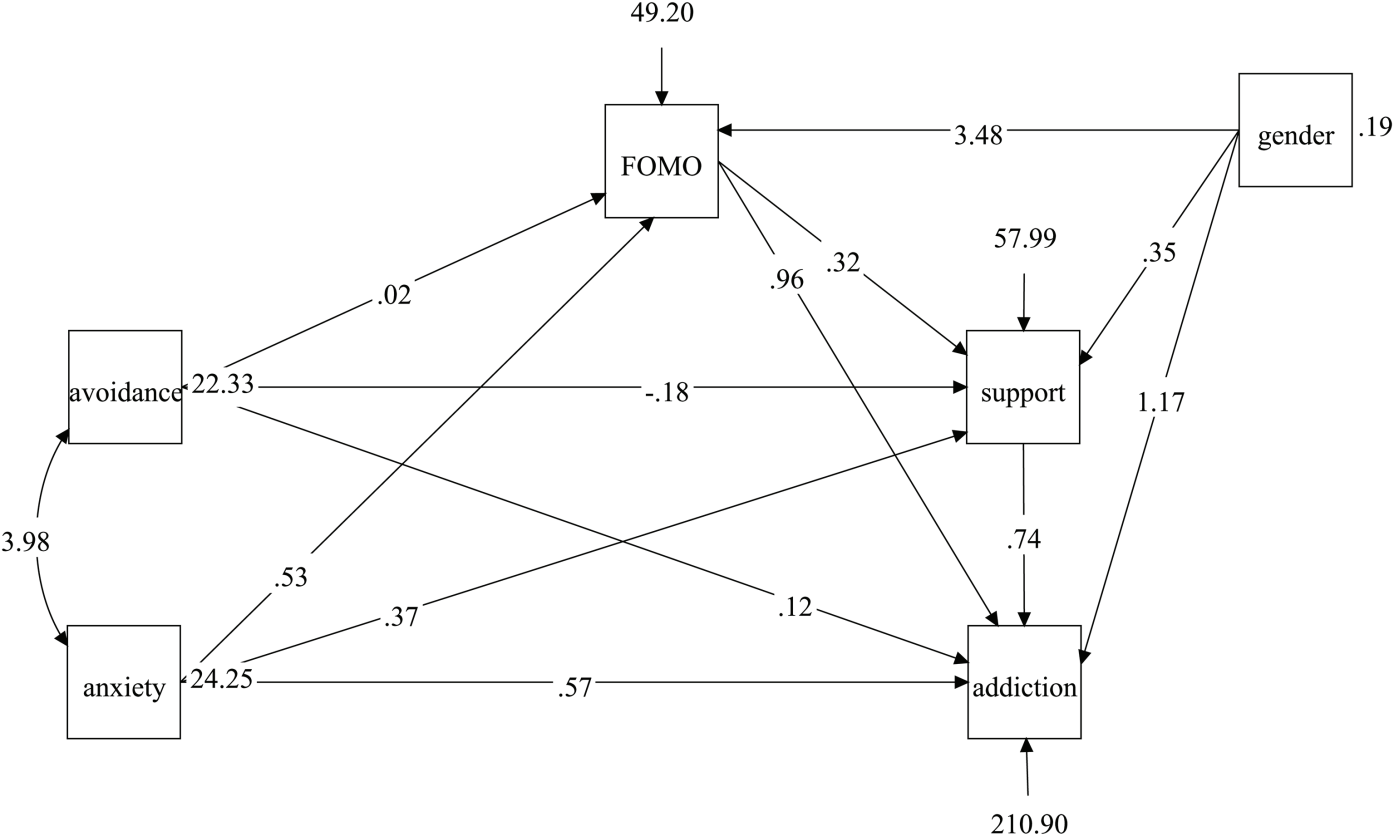
The unstandardized coefficients of the model. All the paths were significant, except the path from avoidant attachment to FOMO and SNS addiction. Gender was added as a control variable.

We estimated the indirect effects of the need for online social support and FOMO on the relationships between insecure attachment and SNS addiction. The results showed that all the paths were significant, except the path from avoidant attachment to FOMO and SNS addiction (*b* = 0.02, *p* = 0.77 and *b* = 0.12, *p* = 0.55, respectively). The whole model showed excellent fit indices [*χ*^2^ = 3.63, *p* of *χ*^2^ = 0.16, CFI = 0.99, TLI = 0.99, RMSEA = 0.04, 95% CI = (0.00, 0.11), SRMR = 0.02]. Specifically, avoidant attachment negatively predicted the need for online social support (*b* = −0.18, *p* < 0.05), while the latter positively predicted SNS addiction (*b* = 0.74, *p* < 0.001). An anxious attachment dimension positively predicted the need for online social support (*b* = 0.37, *p* < 0.001), FOMO (*b* = 0.53, *p* < 0.001), and SNS addiction (*b* = 0.57, *p* < 0.01). Moreover, FOMO positively predicted the need for online social support (*b* = 0.32, *p* < 0.001). Unstandardized coefficients of the model are shown in Figure 2.

The indirect effects of needing online social support and FOMO are shown in Table 2, which indicates that needing online social support negatively mediated the relationship between avoidant attachment and SNS addiction, whereas FOMO, as well as needing online social support, serially and in parallel mediated the relationship between anxious attachment and SNS addiction.

**Table 2:** Indirect effect between insecure attachments and SNS addiction (*N* = 463).

Indirect effects path	Estimate	95% CI	SE	*p*
Total indirect effects from anxiety to SNS addiction	0.92	0.66–1.19	0.14	<0.001
Anxiety → online social support →SNS addiction	0.28	0.11–0.46	0.09	<0.01
Anxiety → FOMO →SNS addiction	0.51	0.36–0.67	0.08	<0.001
Anxiety →FOMO → online social support →SNS addiction	0.13	0.07–0.21	0.04	<0.001
Total indirect effects from avoidance to SNS addiction	−0.02	−0.34 to 0.10	−0.98	=0.33
Avoidance → online social support →SNS addiction	−0.03	−0.27 to −0.02	0.02	<0.05
Avoidance→ FOMO →SNS addiction	0.01	−0.11 to −0.14	0.02	=0.78
Avoidance →FOMO → online social support →SNS addiction	0.01	−0.03 to 0.04	0.28	=0.78

## Discussion

The present study aimed to explore the association between insecure attachment and SNS addiction and test whether both the online social support and FOMO mediate their relationship. We hypothesized that an attachment anxiety would positively predict SNS addiction, while an attachment avoidance would be negatively correlated with SNS addiction. Additionally, we hypothesized that both the need for online social support and FOMO would mediate this association both in parallel and serially. Our results partially supported our hypotheses. Specifically, an anxious attachment dimension positively correlated with SNS addiction, but the link between an avoidant attachment dimension and SNS addiction was not significant. Furthermore, the need for online social support and FOMO mediated the association both in parallel and serially for attachment anxiety, while only online social support mediated this association for an attachment avoidance.

### The Association Between Insecure Attachment Orientation and Social Networking Site Addiction

An anxious attachment orientation is characterized by devoting much time to relationships concerns and a lack of belonging. This can be satisfied by using SNS, which, in turn, may develop into SNS addiction. Conversely, an avoidant attachment dimension is linked with refusal of intimacy and keeping emotional distance in relationships; therefore, individuals with this attachment may use SNS less than other people. As a new type of behavioral addiction, SNS addiction has received much scholarly attention globally in recent years, and there are numerous studies exploring the risk factors linked to SNS addiction (Marino et al., 2018a,b). Prior studies found that attachment, especially insecure attachment, is closely related to SNS general use and addiction (Blackwell et al., 2017; Monacis et al., 2017; Flynn et al., 2018; Worsley et al., 2018a,b; Chen, 2019; D’Arienzo et al., 2019; Marino et al., 2019). Our results are consistent with prior findings. For example, individuals with high attachment anxiety used Facebook more frequently were more likely to use it when feeling negative emotions and were more concerned about how others perceived them on Facebook. While attachment anxiety predicted more feedback seeking and general Facebook activity, attachment avoidance predicted less, and feedback sensitivity mediated the effect of attachment anxiety on Facebook activity (Hart et al., 2015).

### The Mediating Role of Fear of Missing Out and Online Social Support

Beyond examining the association between insecure attachment and SNS addiction, we further tested whether online social support and FOMO mediated this connection. For anxiety attachment, both the need for online social support and FOMO mediate the association in parallel and serially. That is, the more attachment anxiety one feels, the higher his or her FOMO and need for online social support, which, in turn, can lead to a higher level of SNS addiction. This correlation between attachment and FOMO coincided with findings in a previous study (Blackwell et al., 2017). This study also found that FOMO and anxiety attachment were significantly correlated. We hypothesize that it is a basic human desire to have our psychological needs met that links an anxious attachment with FOMO. High attachment anxiety stems from negative interactions in childhood with an unavailable and unresponsive caregiver, in which an individual’s basic psychological needs were neglected and not satisfied. According to the source review of FOMO (Przybylski et al., 2013), if an individual’s need is not satisfied, they can develop FOMO, which can lead to excessive use of SNS. However, the present study did not consider the variable of basic psychological need satisfaction, and future studies could test this assumption. Moreover, an anxious attachment dimension predicted a high level of online social support. Previous studies confirmed the association between anxiety attachment and both perception and provision of social support in offline settings (Collins and Feeney, 2004). The present study added to the current body of literature by showing that individuals with an anxious attachment dimension may also seek support and comfort through SNS to the point of overuse. The present study also found that FOMO positively predicted the need for online social support. Thus, FOMO and the need for online social support serially mediate the relationship between anxious attachment and SNS addiction.

However, for avoidant attachment dimension, only the online social support negatively mediated the avoidance-SNS addiction relationship; the serial mediating effect of FOMO and the need for online social support was not significant. Avoidant attachment is characterized by excessive self-reliance, an inhibition of needs, and avoiding intimacy. Thus, individuals with this attachment may regard online social support as useless, as they believe that there is nobody who would provide help. Moreover, attachment avoidance did not predict FOMO. Although there is evidence that attachment avoidance is correlated with the extent to which basic psychological need is satisfied (Wei et al., 2005), the present study found that attachment avoidance is not correlated with FOMO. These findings indicate that FOMO may stem from attachment anxiety rather than avoidance. Therefore, SNS may not be a useful platform for them to rely on, to keep an eye on what others is doing, and consequently, avoidant attachment is not positively associated with SNS addiction.

### The Theoretical and Practical Implication of Present Study

The present study has both theoretical and practical implications. Theoretically, the present study contributes to the field by showing how insecure attachment is linked to SNS addiction. Although there are several existing studies exploring the association between attachment and SNS addiction, the present study examined the role of online social support and FOMO in this association. In the era of SNS, online social support such as likes, comments, posts, and reposts is so common that its fundamental characteristics are not fully understood. We found that online social support is indeed a risk factor for SNS addiction. Moreover, FOMO has been consistently found to be a predictor of SNS addiction. In the present study, we provide evidence that SNS addiction is correlated with insecure attachment, especially anxious attachment dimension. Practically, these findings could aid in future studies on SNS addiction prevention and interventions. It has been documented that attachment security priming is an effective procedure to reduce attachment anxiety (Gillath and Karantzas, 2019). It entails exposing individuals to stimuli designed to activate a sense of love, comfort, and safety. Attachment security priming may have beneficial effects, especially for those whose attachment involves hypervigilance to threats. Consequently, such a technique could be a potential choice when considering an intervention of SNS addiction. Furthermore, offline social support is important to individuals. If the need for this support is satisfied, they may not turn to SNS for online social support, consequently leading to less SNS use.

### Limitation and Future Directions

The present study is not without limitations. First, this study’s population sample mostly included females (74%), and although we controlled for the effects of gender, this lack of gender balance should be avoided in future research. Second, the study’s cross-sectional design prevented the ability to make causal inferences. Third, the self-report data might threaten the accuracy of the statistical relationships between variables. Last, it is suggested that FOMO may be one dimension of SNS addiction (Kuss and Griffiths, 2017). Thus, future study should test this assumption. However, despite its limitations, the present study demonstrated that attachment theory is useful for explaining addictive behavior relating to SNS. Different from general internet addiction, SNS addiction is a relationship-centered disorder. There are still many variables associated with attachment that need to be further tested in future studies, such as security, emotion regulation strategies and efficacy, psychological distress, and reassurance.

## Conclusions

While an attachment anxiety was significantly and positively correlated with SNS addiction, attachment avoidance was not significantly linked with SNS addiction. FOMO and online social support, in parallel and serially, mediated the relationship between anxiety attachment and SNS addiction, and the need for online social support negatively mediated the relationship between an attachment avoidance and SNS addiction.

## Data Availability Statement

The datasets generated for this study are available on request to the corresponding author.

## Ethics Statement

This study was carried out in accordance with the recommendation of the University’s Human Research Ethics Committee with written informed consent from all subjects. All subjects gave written informed consent in accordance with the Declaration of Helsinki. The protocol was approved by the University’s Human Research Ethics Committee.

## Author Contributions

CL designed the study and collected and analyzed the data. J-LM wrote the manuscript.

### Conflict of Interest

The authors declare that the research was conducted in the absence of any commercial or financial relationships that could be construed as a potential conflict of interest.
